# Pharmacologic RNA splicing modulation: a novel mechanism to enhance neoantigen-directed anti-tumor immunity and immunotherapy response

**DOI:** 10.1038/s41392-021-00789-9

**Published:** 2021-10-28

**Authors:** Kerryn Elliott, Jonas Nilsson, Jimmy Van den Eynden

**Affiliations:** 1 Department of Medical Biochemistry and Cell Biology, Institute of Biomedicine, Sahlgrenska Academy at University of Gothenburg, Gothenburg, Sweden; 2 Sahlgrenska Cancer Center, Department of Surgery, Institute of Clinical Sciences, University of Gothenburg and Sahlgrenska University Hospital, Gothenburg, Sweden; 3 Harry Perkins Institute of Medical Research, University of Western Australia, Perth, WA Australia; 4 Department of Human Structure and Repair, Anatomy and Embryology Unit, Ghent University, Ghent, Belgium

**Keywords:** Immunotherapy, Drug development

A recent study by Lu et al.^1^ suggests a novel approach to increasing responsiveness to immune checkpoint blockade (ICB) therapy. ICB is a promising form of cancer immunotherapy that aims to boost the anti-tumoral immune response. This response is primarily driven by the presentation of different types of antigens at the cancer cell membrane via the type I major histocompatibility complex (MHC I). Neoantigens are tumor-specific antigens that are small (mostly 9-mers) mutated peptides that generally result from somatic mutations. High tumor mutational burden, which results in the formation of many neoantigens, is currently one of the main biomarkers for ICB response prediction. Because ICB is only effective in a minority of patients, new therapeutic strategies are required to augment this response.

The authors of the study, published in *Cell*,^1^ provide preclinical evidence that pharmacological modulation of the spliceosome results in the generation of a substantial amount of highly immunogenic, splicing-derived neoantigens, augmenting the immune response in mice following ICB treatment. If translated to the clinic, these findings raise the exciting possibility of treating otherwise unresponsive cancers with immunotherapy, previously only effective in cancers with increased mutation burden such as melanoma.

The authors explored the effects of two splicing modulating drugs: indisulam and MS-023. The former was previously shown to degrade the accessory splicing factor RBM39, while the latter inhibits type I protein arginine methyltransferase (PRMT) enzymes. They elegantly demonstrate that these drugs have little effect on cancer cell viability in a petri dish, but result in a profound suppression of tumor growth following engraftment into mice. This discrepancy hinted towards an anti-cancer immune response and further experiments using T cell depletion and *B2m* knock out tumor cells confirmed that this drug-induced cancer growth impairment was dependent on T cells and MHC I peptide presentation. Since the compounds did not have an effect on cancer cells themselves, it would have been interesting to see if a CRISPR-mediated knockout of, e.g., RBM39 would result in a viable cell line. If so, the RBM39 null cells could be investigated to determine if they displayed inferior growth compared to an unaltered line in vivo because of T cell immunity.

To determine whether splicing modulation could enhance ICB therapy responses, the authors engrafted mice with syngeneic tumors, followed by pretreatment with splicing inhibitors starting on day 3 and treatment with anti-PD1 on day 7. A significant reduction of tumor growth was detected for simultaneous indisulam (or MS-023) and anti-PD1 therapy, which exceeded the effect of either drug alone. This response was seen for different engrafted tumor cell types (B16-F10 melanoma, MC38 colon carcinoma and LLC lung cancer). LLC was particularly interesting as this cell type is resistant to anti-PD1 monotherapy, yet combination therapy with splicing modulation significantly reduced tumor growth. The anti-tumoral responses resulted in improved survival as compared to mono treatment, with 50% of the mice engrafted with MC38 and treated with MS-023 and anti PD-1 being alive and tumor-free after 3 months. These mice also responded to an additional challenge with MC38 cells 6 months later, suggesting these mice retain immune memory. No toxicity or inflammatory changes were observed in non-tumor tissues. It is worth noting that MC38 cells are generally sensitive to ICB therapy and the other models did not have as great a response.

**Fig. 1:**
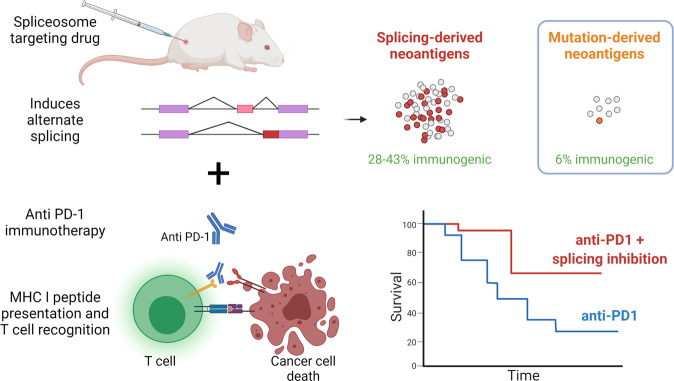
Pharmacologic splicing modulation generates large numbers of highly immunogenic neoantigens which enhance immunotherapy-induced cancer cell death and survival. Box shows estimated amounts of mutation-derived neoantigens for comparison. Created with BioRender.com

The authors then explored the molecular mechanisms behind these promising results. As expected, they identified several splicing alterations with intron retention and exon skipping the most common alterations. Using a combination of in silico MHC binding predictions and mass spectrometry, they demonstrate that these alterations result in a substantial amount of MHC I binding peptides. Interestingly, they confirm that a rather large proportion (up to 43%) of these putative neoantigens are immunogenic, resulting in neoantigen-specific CD8+ T cell activation (Fig. 1).

Altering neoantigen production at the RNA level through splicing modulation has several advantages. Firstly, compared to the alternative, DNA-directed strategies, changes are transient and there is no risk of permanently altering the genetic material of healthy cells. However, the impact of the sustained suppression of RBM39 likely needed to achieve an effect in humans, remains to be tested. Secondly, splicing modulation has the potential to generate thousands of neoantigens, in contrast to the rather limited amount of canonical neoantigens, resulting from somatic mutations. Lastly and most importantly, while these canonical neoantigens are largely similar to their wild-type cognates (e.g., only one out of nine amino acids are different in a 9-mer), splicing-derived peptides are often completely novel to the immune system, resulting in much better immune responses, as illustrated in the study (28–43% of predicted splicing-derived neoantigens were immunogenic versus an estimated amount of 6% for mutation-derived neoantigens^2^) (Fig. 1).

It remains to be determined whether the approach will result in direct clinic applications. While the authors did not observe any toxicity in their mice models, inflammatory side effects could be a major clinical concern, especially when combined with ICB. In humans, drug treatments usually continue for several months. Since splicing inhibition could have the risk of causing side-effects such as developmental defects^3^, safety needs to be carefully monitored in additional species than mice before clinical trials are initiated. Further, clinical effectiveness is uncertain as several immune evasion mechanisms have been described in human tumors that could interfere with (splicing-induced) neoantigen presentation (e.g., *B2M* mutations or *HLA* loss). Relatedly, it is also unclear from the study whether splicing modulation can induce long-lasting immune responses. In this regard, it remains to be established whether memory T-cells are raised against these splicing-derived neoantigens and whether any reaction is still possible after the removal of the splicing inhibitor.

Apart from its direct translational and clinical relevance, the findings provide important insights in cancer biology and tumor evolution. Assuming that splicing inhibition could indeed be a powerful source of highly immunogenic neoantigens, strong negative selective forces are expected against genomic alterations in the genes encoding the targeted proteins. This sheds new light on the interpretation of previously detected negative selection signals in RNA splicing genes.^4^ The study also hints at a largely underappreciated role of the noncoding genome in neoantigen formation and is in line with the work of Laumont et al.^5^ who demonstrated that 90% of tumor-specific antigens are derived from noncoding regions.

In conclusion, the study of the groups of Abdel-Wahab and Bradley provides important insights in cancer immunobiology with direct clinical relevance. Further translational and clinical research is required to demonstrate whether pharmacologic splicing modulation is a realistic treatment option in a clinical cancer immunotherapy setting.
